# Relationship between attentional bias and psychological craving in methamphetamine use disorder

**DOI:** 10.3389/fpubh.2025.1659759

**Published:** 2026-01-05

**Authors:** Qiuping Huang, Zhenjiang Liao, Xuhao Wang, Wenwu Wang, Li Chao, Yiqi Nie, Shihua Peng, Lin Zhao, Hongxian Shen, Jing Qi, Xinxin Chen

**Affiliations:** 1 Department of Psychology, School of Humanities and Management, Hunan University of Chinese Medicine, Changsha, Hunan, China; 2 Department of Psychiatry, National Clinical Research Center for Mental Disorders, The Second Xiangya Hospital of Central South University, Changsha, Hunan, China; 3 Hunan Provincial Lushan Compulsory Isolation Detoxification Center, Changsha, Hunan, China; 4 The Second People’s Hospital of Hunan Province (Brain Hospital of Hunan Province), Changsha, Hunan, China

**Keywords:** methamphetamine use disorder, attentional bias, cue-induced craving, Stroop task, withdrawal craving

## Abstract

**Background:**

Psychological craving and attentional bias are important indicators of addiction, as well as critical factors influencing relapse. Psychological craving includes withdrawal craving (a persistent background state during abstinence) and cue-induced craving (an acute, impulsive desire provoked by drug-related stimuli). However, the relationship between attentional bias and these two types of craving remains unclear. This study aims to investigate the association between attentional bias and psychological craving in individuals with methamphetamine use disorder (MUD) using a picture-based addiction Stroop task.

**Methods:**

A total of 134 individuals with MUD were recruited. General demographic information and details regarding substance use were collected through questionnaires. For withdrawal craving, the visual analog craving scale (VAS) was used to assess the level of drug craving during the abstinence period. To assess cue-induced craving, participants were presented with drug-related scenarios through virtual reality (VR). Craving was then measured in each virtual scenario using a visual analog scale (VAS) integrated into the system. Subsequently, attentional bias was evaluated by the picture addiction Stroop task.

**Results:**

Methamphetamine use disorder participants demonstrated significantly longer reaction times to MA-related image stimuli compared to neutral image stimuli (*p* < 0.001). Attentional bias scores were positively correlated with both withdrawal craving (*p* = 0.001) and cue-induced craving (*p* = 0.043). Significant differences in attentional bias scores were observed among groups with varying levels of withdrawal craving (*F* = 5.364, *p* = 0.006). Multiple linear regression analysis revealed that withdrawal craving was independently and specifically associated with attentional bias (*β* = 8.135, *p* = 0.006).

**Conclusion:**

Individuals with MUD exhibit significant attentional bias toward MA-related cues, which is associated with the intensity of both withdrawal and cue-induced craving. Furthermore, attentional bias is significantly associated with withdrawal craving rather than cue craving. These findings provide a foundation for developing interventions targeting attentional bias and craving in MUD.

## 1 Introduction

Psychological craving, a subjective motivational state (1), refers to an intense urge to re-experience the effects of psychoactive substances (2, 3). It persists long after withdrawal and is significant in diagnosis, prognosis, and determining clinical efficacy (4–6). Dysfunctional craving mechanism is considered a primary etiological factor contributing to the relapse (7–9). Galloway et al. found that psychological craving predicts MA reuse during treatment, with the highest prediction accuracy at 1 week and declining accuracy with longer evaluation periods (10). Despite some debate (11), craving is generally accepted as a predictor of relapse (10, 12). Craving occurs in two distinct forms, withdrawal craving and cue-induced craving (13). Often called background craving, withdrawal craving is an endogenous state inherently linked to physiological withdrawal (1, 14). Withdrawal craving is primarily regulated by the extended amygdala-lateral habenula-nucleus accumbens circuit, characterized by reward deficiency and excessive stress system activation (15). It relies on elevated stress neurotransmitters [e.g., corticotropin-releasing factor (CRF), dynorphin] in the extended amygdala and reduced dopamine release in the nucleus accumbens, which jointly induce negative emotions like anxiety and anhedonia, driving drug-seeking to alleviate suffering (16, 17). Meanwhile, lateral habenula activation further inhibits the midbrain dopamine system, worsening reward deficits (18). By contrast, cue-induced craving is situation-specific, triggered mainly by MA use-related environmental cues—such as drug paraphernalia (e.g., syringes, lighters, straws) or specific social contexts (19, 20). It is mediated by the prefrontal cortex-basal ganglia-insula circuit, centered on enhanced motivational salience from drug-related cues: the prefrontal cortex activates dopamine release in the basal ganglia (ventral striatum) via glutamatergic projections to convert cues into strong reward signals; the insula transforms subconscious motivation into explicit subjective craving through interoceptive function; meanwhile, prefrontal executive dysfunction impairs the inhibition of drug-seeking behavior (15).

Attentional bias is defined as difficulty in controlling attention to drug-related cues, which impairs performance on the current task. This bias plays a pivotal role in triggering cravings for addictive substances and initiating automatic drug-seeking behaviors (21). Moreover, it serves as an important behavioral reaction indicator in cue reactivity (22, 23). A key factor in attentional bias is impaired cortical structure and function from long-term chronic use of addictive substances, primarily manifested in the lateral prefrontal cortex and dorsal anterior cingulate gyrus (24, 25).

Common paradigms used to study attentional bias include the dot-probe task and the Stroop task. The Stroop task is relatively simple to administer and provides a better reflection of an individual’s attentional control (26). In Stroop tasks, most studies use MA-related vocabulary, including terms related to tools, scenarios, and motivations. Attentional bias in individuals with MUD is explored via reaction times in color-naming tasks. However, research suggests vocabulary stimuli have limited ecological validity and cannot sensitively detect addiction severity (27). Additionally, pictures elicit stronger priming effects than words when used as initiating stimuli (28). Thus, improving the ecological validity of stimulus materials is necessary to explore the impact of the Addiction Stroop effect on MUD.

Attentional bias is widely recognized as closely related to craving. Franken suggests a bidirectional causal relationship between attentional bias and craving (29). Specifically, increased attentional bias leads to increased subjective craving and subsequent drinking behavior (30, 31). And as craving increases, substance-related stimuli become more pronounced. However, experimental decreases in attentional bias did not appear to correspond to reductions in craving or drinking behavior (32). Therefore, it is crucial to investigate the relationship between attentional bias and motivational states such as craving (33), which could inform effective clinical interventions (21).

Several models have examined the association between craving and attentional bias, among which the incentive-sensitization theory stands as a representative (21). As Robinson and others argue, substance-related cues possess the characteristic of incentive sensitization. The core principle of this theory is that repeated substance use triggers dopaminergic responses, which become increasingly sensitive with each episode of use (34). This process endows addicts with stronger motivational attributes, thereby enhancing their subjective craving for the substance. Through classical conditioning, substance-related cues acquire these motivational properties, diverting attention toward themselves, making them attractive and “wanted, “and thus guiding behavior toward the motivation (34). Therefore, this model suggests that subjective craving and attentional bias may share an underlying mechanism, implying a correlation between the two. However, researchers have also pointed out that the motivational properties of substance-related cues can drive substance-seeking behavior unconsciously, which may mean that subjective craving and attentional bias can be dissociated in certain situations (34). Franken’s study (33) supports the incentive-sensitization theory, holding that after extensive experience with substance use, substance-related cues can capture the attention of substance users as a result of cue-induced dopamine release in the corticostriatal circuit (35).

Other models based on cognitive psychology have made similar assumptions. For example, Ryan suggests that cue reactions and craving experiences are associated with perceptual and cognitive processes that occur before, during, and after cue exposure (36). This model argues that substance-related stimuli receive prioritized attentional processing, which is a key determinant of the subjective craving in response to cues. Increased craving heightens attention to substance-related cues, and vice versa (21). Kavanagh et al. (37) proposed the intrusion theory of desire, which states that subjective craving may initially be an intrusion, potentially triggered by internal states (such as withdrawal symptoms) or external cues (such as seeing someone using the substance). Once drug users become aware of this craving, they ruminate on the craving itself or sustain attention on the external cues that triggered it (37). This rumination, in turn, intensifies the strength of subjective craving (37). Hence, this model also implies a bidirectional causal relationship between the selective attentional processing of substance-related cues and subjective craving.

Building on the above research background, this study developed a picture-based MA addiction Stroop paradigm. By measuring MUD participants’ reaction times to MA-related and neutral images, this study aims to explore the behavioral characteristics of their attentional bias. Our prior research identified a close relationship between withdrawal craving and cue craving, both associated with the severity of MUD. Given that withdrawal craving and cue-induced craving diverge fundamentally in their triggering sources (endogenous physiological withdrawal and exogenous environmental cues) and neural regulatory circuits, their motivational underpinnings that drive attention allocation may also differ. The existing theoretical models have primarily focused on generalizing the link between overall craving and attentional bias, and have not yet fully explored the potential differential associations between these two craving subtypes—distinct in function and mechanism—and attentional bias. This relative lack of targeted investigation highlights the necessity of specifically exploring how withdrawal craving and cue-induced craving may, respectively, relate to attentional bias in MUD participants. Building on this, the present study further investigates the relationship between attentional bias and these two types of psychological craving in MUD participants. Two hypotheses are proposed: First, MUD participants will exhibit significant attentional bias toward MA-related images versus neutral images. Second, their attentional bias will be significantly correlated with both withdrawal craving and cue-induced craving. Furthermore, given the compelling nature of withdrawal-related negative reinforcement, we exploratorily hypothesized that the association with withdrawal craving might be particularly pronounced.

## 2 Materials and methods

### 2.1 Participants

In this study, a total of 150 individuals with MUD were recruited from a compulsory drug rehabilitation center in China. Participants were required to meet the following inclusion criteria: (1) Meet the DSM-5 criteria for stimulant use disorder; (2) Have used MA at least twice a month for six consecutive months within the past 2 years (counting from the admission date to the rehabilitation center); (3) Be male, aged 18–55, and had normal color vision as confirmed by the Ishihara test; (4) Have an education level above primary school. The exclusion criteria were as follows: (1) have a history of polysubstance abuse (counting from the admission date to the rehabilitation center); (2) have a personal or family history of mental illness, or severe physical diseases.

This study was approved by the Ethics Review Committee of the Second Xiangya Hospital of Central South University in 2019, with the approval number (2019) Ethical Review [Science] No. (071). Each participant personally signed a paper-based informed consent form.

### 2.2 Research tools

#### 2.2.1 Demographic variables and substance use

Demographic variables included age, occupation, education level, marital status, residence, monthly income, and permanent members of the household. The consumption and usage patterns of MA, tobacco, and alcohol among participants was evaluated. The assessment of MA use included the following aspects: age of first drug use, duration of MA use (in months), length of abstinence (in months), dose of MA (g per use), frequency of MA use in the year before abstinence, frequency of MA use in the month before abstinence, and number of compulsory drug detoxifications. The Fagerstrom Test for Nicotine Dependence (38) (FTND) and the Alcohol Use Disorder Identification Test (39) (AUDIT) were employed to assess tobacco and alcohol use, respectively.

#### 2.2.2 Withdrawal craving and cue-induced craving

Withdrawal craving, defined as the participants’ current level of craving for MA during the withdrawal period (13), was assessed using the Visual Analogue Scale (VAS). A score of 1 on the VAS indicates the complete absence of craving, while a score of 10 represents an extremely intense craving (40). Higher scores are associated with a stronger subjective level of craving.

Cue-induced craving was evaluated using a virtual reality (VR) cue-exposure paradigm preliminarily developed by our research team (20). It contained four VR scenes, including a resting scene (with guiding audio and a system logo interface audible only in the first 10 s), two neutral scenes (featuring underwater and elephant-walking grassland scenes, with natural sounds such as wind and underwater noises), an MA-item scene (presenting static cues such as MA and drug-using paraphernalia), and a drug-using scene (showcasing dynamic cues of the social context where an actor is using MA). Our previous study showed that drug-using scene could induce stronger cue-induced craving (20). Therefore, in the present study, the craving scores in response to the VR drug-using cues were used as the indicator of cue-induced craving for further analysis.

#### 2.2.3 Attentional bias

Attentional bias was evaluated using a self-designed image-based addiction Stroop task paradigm.

##### 2.2.3.1 Task material

The MA-related cue images and neutral cue images were included in this self-administered picture addiction Stroop task paradigm. It contained 30 MA-related images and 30 neutral images, and each image was unique. These pictures were selected in our previous research (41). All MA-related images were authentic and shot by researchers using a camera. Drug paraphernalia and simulated drug-use scenarios were self-developed and modified after testing by MUD individuals. Each MA-related image was scored subjectively by MUD individuals, and 30 of the 150 images were selected by score. Neutral cue images were taken from high-resolution images and photographs on the Internet, including everyday objects (e.g., lunch boxes and buttons) and action behaviors (e.g., mopping floors and washing dishes). The pictures were processed uniformly using Photoshop. The characters in the pictures were mosaic processed, the color difference was removed, and black and white processing was carried out. All the pictures had the same size (320*240 pixels), resolution (72 ppi), contrast, brightness, and other physical attributes. The MA users have rated all the pictures from three perspectives: clarity, simulation, and content. Both MA cue pictures and neutral pictures have good reliability (41).

##### 2.2.3.2 Task design

There were two levels of picture types, MA-related picture stimuli and neutral picture stimuli. Each picture had squares of different colors (red, yellow, blue, green, 12*12 pixels) that appeared pseudo-randomly at the top and bottom of the picture. Consequently, there were a total of 240 stimuli. The presentation order of the 240 stimuli was pseudo-random, following two core principles: (1) to prevent sequence effects, small squares of the same color were not allowed to appear consecutively more than twice; (2) the distribution of MA-related and neutral picture stimuli was balanced throughout the experiment—neither picture type was concentrated in any segment, while the overall proportion of the two types remained equal across all stimuli. Each stimulus is presented for 4,000 ms, with the response window strictly coinciding with this 4,000 ms presentation period—participants can only respond while the stimulus is visible. The inter-stimulus interval is randomly set between 500 ms and 1,500 ms. E-Prime 2.0 software was used to present the pictures.

##### 2.2.3.3 Task procedure

The experimenter instructed the participants to place their hands naturally on the keyboard, with the index and middle fingers of the left hand on the reaction keys F and D, respectively, and the index and middle fingers of the right hand on the reaction keys J and K, respectively. In each round of the test, the background picture and the small square appeared simultaneously, and participants made a key press reaction according to the color of the small square. The color-key correspondences were as follows: press D for red, F for green, J for yellow, and K for blue. Participants were asked to respond as accurately and quickly as possible, i.e., to press the keys as quickly as possible while ensuring accuracy. The correspondence between colors and response keys was applied to all subjects to avoid inter-individual variability caused by different key mappings. The entire task consisted of a practice phase and a formal phase. The small square randomly appears at the top and bottom positions of a gray blank image in the practice phase. The purpose of the practice phase was to establish the link between the colors and the key presses through random presentations of the color blocks. After completing the practice module, participants were instructed to press the key Q to begin the formal task. They were allowed to take a short break after completing 120 reaction and they could press the key Q to continue when they felt ready. The total experiment time was approximately 20 min.

### 2.3 Research procedure

This was a cross-sectional study conducted from April 2019 to June 2019. Participants were screened in accordance with the inclusion and exclusion criteria, and their written informed consent was duly obtained. Subsequently, the researchers conducted interviews and questionnaire assessments with the participants. In a quiet and comfortable treatment room, the participants underwent a 10-min practice session with the VR neutral scenario. After that, during the formal experiment, the subjective craving scores of the participants in the standardized VR scenarios were collected. After the VR assessment, researchers interviewed each participant to understand their states after cue induction. Relaxation training and interventions were provided as needed. Finally, the participants were invited to perform the picture-based addiction Stroop task. The picture-based addiction Stroop task was administered at least 3 days after the VR assessment. To alleviate any potential distress following VR cue exposure, all participants received a standardized, brief (5-min) guided breathing relaxation exercise administered by a trained researcher. The exercise involved instructing participants to close their eyes and focus on taking slow, deep breaths. The same script was used for all participants.

### 2.4 Statistical methods

SPSS 20.0 was used to organize and analyze the data. Descriptive statistics were employed to analyze the general information of the participants, their addiction assessment results, and psychological craving scores. For the attentional bias assessment, data were first screened: participants with less than 90% correct responses in the formal task or reaction times outside 3 standard deviations of the entire sample’s mean were excluded (42). The specific evaluation metrics were the reaction times (in milliseconds) for correct responses to MA-related and neutral pictures. The mean reaction time for proper reactions to each category of picture stimuli was calculated and analyzed with descriptive statistics. Paired-sample t-tests were used to compare reaction times for the two categories of picture stimuli. Pearson correlation analysis was used to correlate attentional bias with psychological craving in people with MUD. The attentional bias score was calculated as the difference between the correct reaction time to the MA-related pictures and that to the neutral pictures. Since the VR drug use scene induces stronger cue craving, in this study, the craving score under the cue of the VR drug use scene was used as the cue craving value for further analysis. For further exploring the relationships among attentional bias, withdrawal craving, and cue craving, the different craving level scores were categorized into three groups based on the distribution of craving scores (full spacing of 1–10 points) by taking the upper and lower percentiles (p33, p66). Thus, withdrawal craving scores equal to 1 were delineated as the no craving group (*n* = 39), 2–3 as the low craving group (*n* = 48), and 4–10 as the high craving group (*n* = 47). According to the VR drug use scenarios, craving scores equal to 1 were delineated as the no cue craving group (*n* = 32), 2–3 as the low cue craving group (*n* = 45), and 4–10 as the high cue craving group (*n* = 57).

A repeated measures ANOVA was used to compare the groups of participants with different levels of withdrawal craving and cue craving in terms of their responses to different stimulus pictures (neutral pictures, MA-related pictures). A one-way ANOVA was used to analyze the groups of participants with different levels of withdrawal craving and cue craving in terms of attention bias. A multiple linear regression analysis was used to identify the effect of cue craving and withdrawal craving on attentional bias. A 95% confidence interval was set, and all statistical tests were two-sided with a significance level of *p* < 0.05. The Pearson correlation analysis was subjected to Bonferroni correction for multiple comparisons, with the significance threshold set as *α* = 0.05/2 = 0.025.

## 3 Results

### 3.1 Participants’ basic information

In this study, we recruited a total of 150 participants with MUD. Of these, 137 completed the addiction Stroop task while 13 did not. Additionally, three participants were excluded due to their accuracy rates in the task below 90% (50, 88.75, and 88.75% respectively). Consequently, the valid data of 134 MUD participants were finally included in this study. Their sociodemographic characteristics and substance use information are summarized below.

#### 3.1.1 Sociodemographic data

Among the 134 MUD participants, the mean age was 36.40 years (SD = 7.69), ranging from 21 to 53 years. A total of 55.2% were unemployed before admission, 60.4% had a middle school education, and 50.0% reported a monthly income between 1,000 and 5,000 yuan. Other sociodemographic variables are detailed in Table 1.

**Table 1:** Demographic information (*N* = 134).

Variable	*M* ± SD	*n*(%)
Age (years)	36.40 ± 7.69	
Pre-rehabilitation occupation		
Yes		60 (44.8%)
No		74 (55.2%)
Education level		
Elementary school		21 (15.7%)
Junior high school		81 (60.4%)
High school/secondary vocational school		32 (23.9%)
Marital status		
Unmarried		45 (33.6%)
Married		50 (37.3%)
Divorced		39 (29.1%)
Residence		
Urban		56 (41.8%)
Town		44 (32.8%)
Rural		34 (25.4%)
Monthly income		
No income		29 (21.6%)
Less than ¥5,000		67 (50.0%)
More than ¥5,001		38 (28.4%)
Permanent members of the household		
0–2		39 (29.1%)
3		45 (33.6%)
4 or more		50 (37.3%)

#### 3.1.2 Substance use

The age of first MA use for the 134 MUD participants was 28.04 years (SD = 8.06, range 13–46 years). The cumulative length of MA use was 57.49 months (SD = 35.25), and the length of consecutive MA abstinence was 18.57 months (SD = 1.65, range 14–23 months). Moreover, the average MA dosage of the participants while regularly using MA was 0.70 grams per use (SD = 0.54). Detailed data are shown in Table 2. Concerning the utilization of other substances, 59.7% of the participants drank alcohol, with an AUDIT score of 12.23 ± 7.27, and 91.8% smoked cigarettes, with an FTND score of 4.56 ± 1.90.

**Table 2:** Substance use status (*N* = 134).

Variable	*M* ± SD	*n*(%)
Age of first drug use	28.04 ± 8.06	
Duration of MA use (months)	57.49 ± 35.25	
Length of abstinence (months)	18.57 ± 1.65	
Dose of MA (g/each)	0.70 ± 0.54	
Frequency of MA use in the year before abstinence		
≥1 time/day		69 (51.5%)
<1 time/day		65 (48.5%)
Frequency of MA use in the month before abstinence		
≥1 time/day		79 (59%)
<1 time/day		55 (41%)
Number of compulsory drug detoxification (times)		
First compulsory detoxification		27 (20.1%)
2 times compulsory detoxification		53 (39.6%)
3–5 times compulsory detoxification		54 (40.3%)
Drinking		
Yes		80 (59.7%)
No		54 (40.3%)
Smoking		
Yes		123 (91.8%)
No		11 (8.2%)
AUDIT Score	12.23 ± 7.27	
FTND Score	4.56 ± 1.90	

### 3.2 Attentional bias characteristics of MUD participants

**Figure 1:**
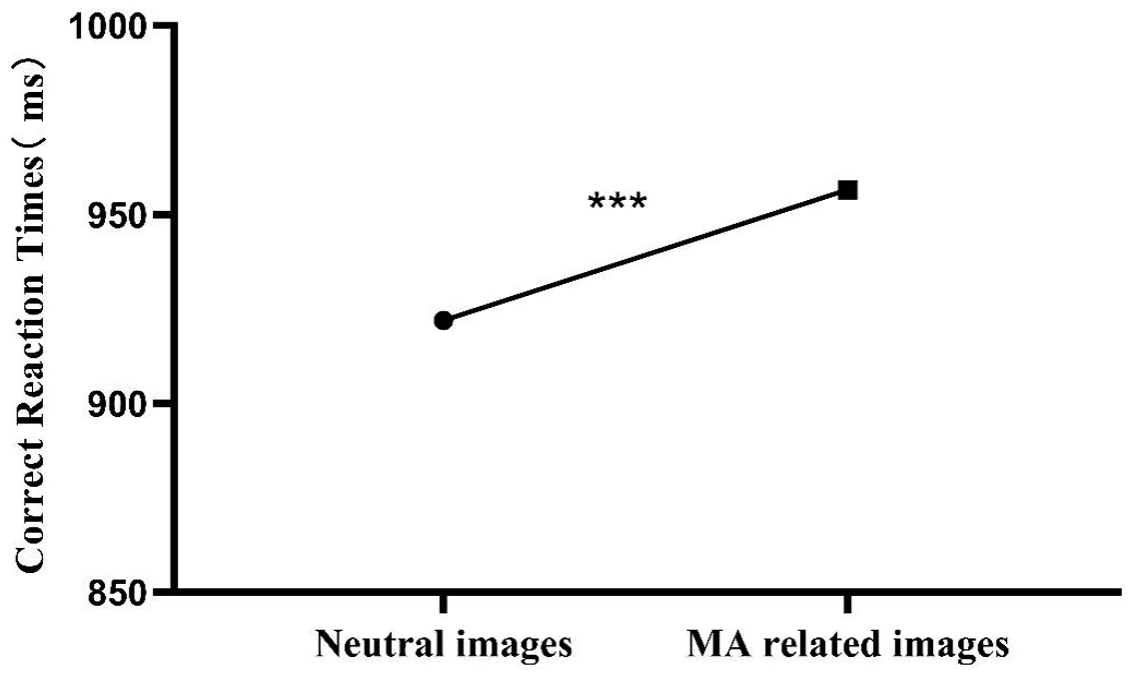
Comparison of correct reaction times to neutral and MA-related images in MUD individuals. ***p < 0.001.

This study used the addiction Stroop task paradigm to assess the attention bias of MUD participants toward MA-related image cues. As shown in Table 3 and Figure 1, participants’ reaction times to MA-related image stimuli were significantly longer than those to neutral image stimuli (*t* = −6.114, *p* < 0.001). This indicates that MUD participants exhibited a pronounced attention bias toward MA-related cues.

**Table 3:** Comparison of reaction times to two types of image stimuli in different craving groups with varying states and degrees of craving.

Different states/degrees of craving groups	Neutral image stimuli	MA-related image stimuli	*F/t*	*p*
Withdrawal craving level				
No craving (*n* = 39)	888.88 ± 128.64	917.35 ± 137.88	7.810	0.006
Low craving (*n* = 48)	953.94 ± 159.17	970.55 ± 166.42	3.272	0.073
High craving (*n* = 47)	916.56 ± 131.14	974.87 ± 179.89	39.483	<0.001
Cue-induced craving level				
No craving (*n* = 32)	902.83 ± 143.25	922.71 ± 147.17	2.951	0.088
Low craving (*n* = 45)	931.11 ± 150.57	964.44 ± 178.78	11.664	0.001
High craving (*n* = 57)	925.32 ± 137.19	969.40 ± 162.09	25.828	<0.001
All participants (*N* = 134)	921.90 ± 142.58	956.58 ± 164.47	−6.114	<0.001

### 3.3 Relationship between attentional bias and craving in MUD participants

#### 3.3.1 Pearson correlation between attentional bias and withdrawal craving, cue-induced craving

Results from Pearson correlations showed that the attentional bias score of MUD participants was positively correlated with withdrawal craving scores (*r* = 0.277, *p* = 0.001) and cue-induced craving scores under VR drug-use scenarios (*r* = 0.175, *p* = 0.043) (Table 4). The correlation with withdrawal craving passed multiple comparison correction, while the correlation with cue-induced craving did not.

**Table 4:** Pearson correlation analysis of attentional bias and psychological craving in MUD individuals.

Different types of cravings	Reaction time to MA-related image stimuli		Reaction time to neutral image stimuli		Reaction time difference	
	*r*	*p*	*r*	*p*	*r*	*p*
Withdrawal craving score	0.120	0.169	0.010	0.906	0.277	0.001
Cue-induced craving score	0.138	0.113	0.078	0.370	0.175	0.043

#### 3.3.2 Reaction time comparisons between MA-related and neutral images among MUD participants at different craving levels

**Figure 2:**
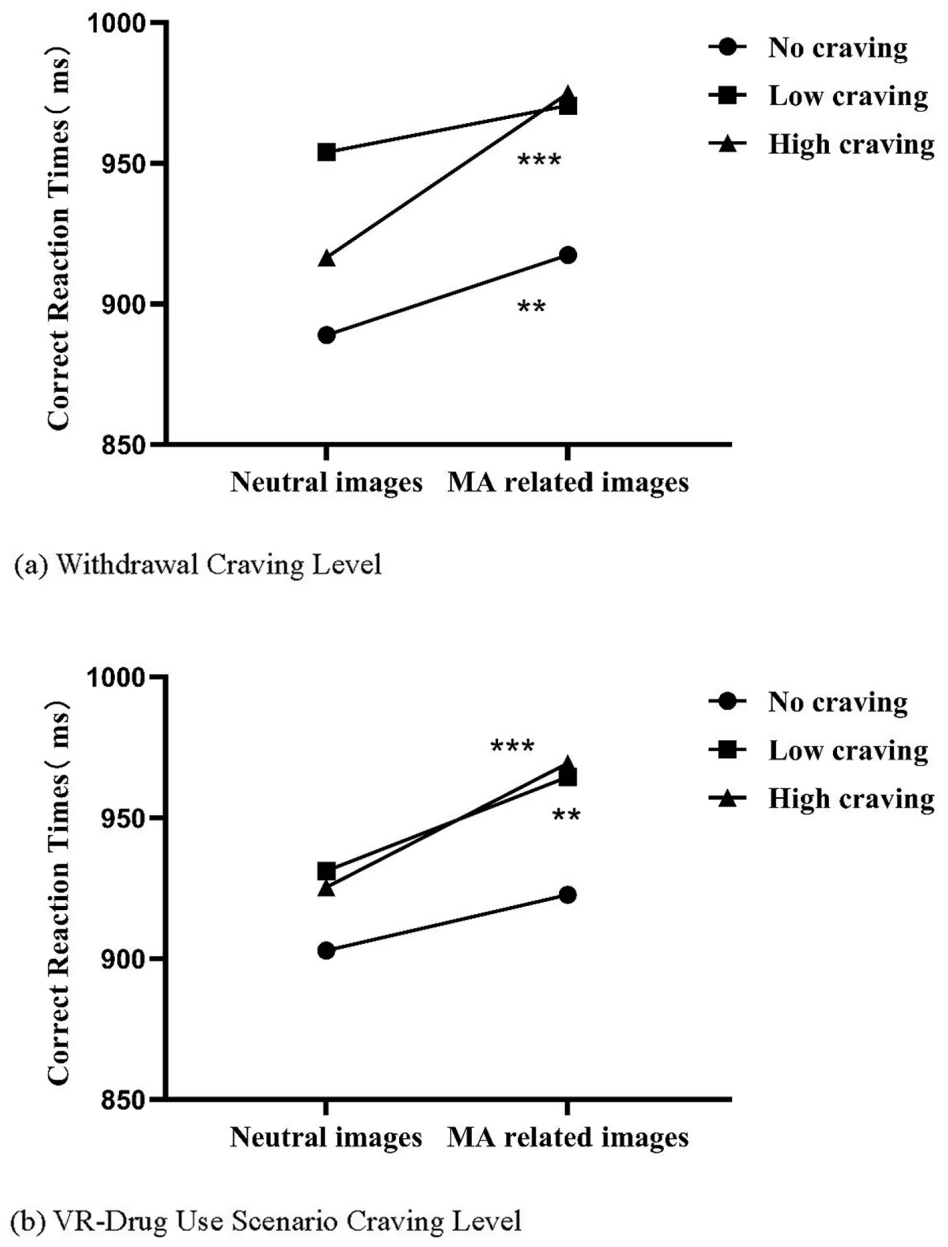
Comparison of correct reaction times for neutral image stimuli and MA-related image stimuli across groups. **(a)** Withdrawal craving level. **(b)** VR-drug use scenario craving level. ***p < 0.001; **p < 0.01.

This study divided participants into three groups based on the distribution of craving score. A 3-group×2-stimulus repeated-measures ANOVA was conducted, with image type as the within-subject factor. Results showed that reaction times to MA-related images were significantly longer than those to neutral images in the no-withdrawal-craving group [*F*_(1,38)_ = 7.810, *p* = 0.006, *η^2^* = 0.056], high-withdrawal-craving group [*F*_(1,46)_ = 39.483, *p* < 0.001, *η^2^* = 0.232], low-cue-craving group [*F*_(1,44)_ = 11.664, *p* = 0.001, *η^2^* = 0.082], and high-cue-craving group [*F*_(1,56)_ = 25.828, *p* < 0.001, *η^2^* = 0.165]. This indicated a significant attentional bias toward MA-related cues. However, no significant differences in reaction times were found between the two images types in the low-withdrawal-craving group [*F*_(1,47)_=3.272, *p* = 0.073, *η^2^* = 0.024] and the no-cue-craving group [*F*_(1,31)_=2.951, *p* = 0.088, *η^2^* = 0.022]. Furthermore, no significant group-level differences were observed across all comparisons (*p* > 0.05). Detailed data are presented in Table 3 and Figure 2.

#### 3.3.3 Comparison of attentional bias scores among MUD participants at different craving levels

**Figure 3:**
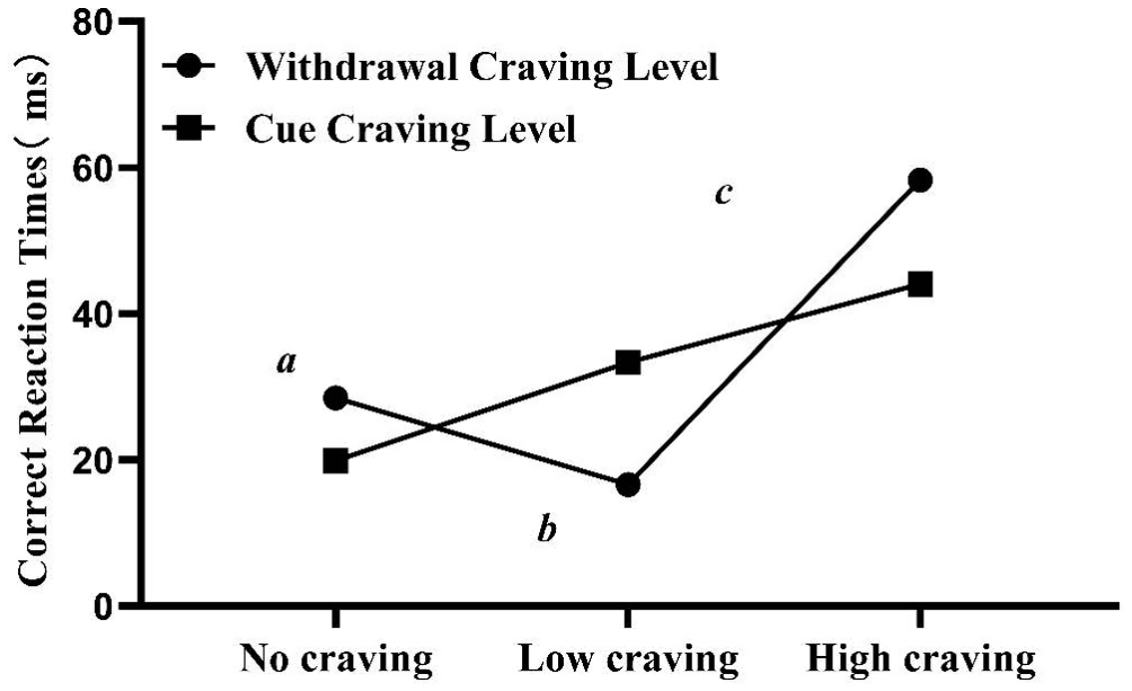
Comparison of attention bias scores among groups with different levels of withdrawal and cue-induced craving.

The one-way ANOVA showed that there were significant differences in attentional bias scores among the no-, low-, and high-withdrawal craving groups (*F* = 5.364, *p* = 0.006). *Post hoc* analyses revealed that the high-withdrawal craving group had significantly higher attentional bias scores compared to both the no-withdrawal craving group (*p* = 0.032) and the low-withdrawal craving group (*p* = 0.002). However, no significant differences were found in attentional bias scores among groups with different levels of cue-induced craving (*p* > 0.05). The detailed results can be found in Table 5 and Figure 3.

**Table 5:** Comparison of attentional bias scores to two types of image stimuli in different craving groups with varying states and degrees of craving.

Different states/degrees of craving groups	Attentional bias scores	*F/t*	*p*	LSD
Withdrawal craving level		5.364	0.006	
No craving (*n* = 39) a	28.47 ± 50.44			a < c, *p* = 0.032 b < c, *p* = 0.002
Low craving(*n* = 48) b	16.61 ± 41.60			
High craving(*n* = 47) c	58.31 ± 87.51			
Cue-induced craving level		1.413	0.247	
No craving (*n* = 32)	19.88 ± 45.22			
Low craving (*n* = 45)	33.33 ± 65.85			
High craving (*n* = 57)	44.07 ± 74.08			

#### 3.3.4 Multiple linear regression analysis results of cue craving and withdrawal craving on attentional bias

To further delineate the unique contribution of each craving type, a multiple linear regression was conducted with attentional bias score as the dependent variable and both withdrawal craving and cue-induced craving scores as independent variables. The overall regression model was statistically significant [*F*_(2, 131)_ = 6.130, *p* = 0.003], indicating that the two craving measures together significantly predicted attentional bias. There was no evidence of multicollinearity (all tolerance value = 0.902, all VIFs = 1.108). Critically, withdrawal craving was a significant unique predictor (*β* = 8.135, *p* = 0.006), whereas cue-induced craving was not (*β* = 2.643, *p* = 0.266). This result confirms that attentional bias is independently and specifically associated with the severity of withdrawal craving, but not with cue-induced craving, after accounting for their shared variance.

## 4 Discussion

Based on the Picture Addiction Stroop task, this study investigated the attentional bias characteristics of male individuals with MUD in long-term abstinence and their relationship with psychological craving. The results revealed that these individuals exhibited a significant attentional bias toward MA-related cues, and this bias varied with craving levels. Individuals in no-, high- withdrawal craving groups and low-, high- cue craving groups displayed significantly higher reaction times to MA-related picture stimuli compared to neutral picture stimuli. Additionally, individuals with different withdrawal craving levels showed significant differences in attentional bias.

### 4.1 MUD individuals exhibit significant attentional bias toward MA-related stimuli

This study found that compared to neutral cues, individuals with MUD in long-term abstinence displayed a significant attentional bias toward MA-related cues. This finding is consistent with previous studies (27, 43–46), which provide strong evidence of attentional bias in various addictive behaviors, including opioid and cocaine use disorders, as well as nicotine and alcohol use disorders (21, 26, 31). Such attentional bias persists across different paradigms used to assess attentional bias (46). The selective attention to MA-related cues among MUD individuals serves as the foundation for this attentional bias. According to incentive-sensitization theory, substance-related cues possess motivational properties and can capture the attention of individuals with MUD. This results in a significant increase in their motivation to obtain the substance, and in turn, triggers drug-seeking behavior (47). From a neurobiological perspective, long-term MA use leads to structural and functional impairments in the brain, particularly in the prefrontal cortex. Damage to this area reduces the ability of individuals with MUD to control attention, making them unable to suppress irrelevant stimuli during tasks (48).

Unlike the traditional Stroop task for vocabulary addiction, the present study used pictures as the priming stimulus. Previous research has found that pictures elicit more priming effects than words (28). This study compared the reaction times (neutral image reaction time: 921.90 ± 142.58 ms, MA-related image reaction time: 956.58 ± 164.47 ms) with those from Zhu’s study (49), which investigated attentional bias in MUD participants using a lexical addiction Stroop task (neutral word reaction time: 723.30 ± 118.56 ms, MA-related word reaction time: 728.47 ± 118.05 ms). It was found that MUD individuals exhibited longer reaction times to images compared to words. Additionally, the variability in image reaction times was higher than that for word reaction times. These results are consistent with Hester et al.’s findings on attentional bias in cocaine users (50). This suggests that MUD individuals’ selectively attend to both neutral and MA-related images, possibly due to their preferences and the inherent characteristics of image and word stimuli. Despite this, the image-based addiction Stroop task remains a sensitive tool for studying attentional bias (50).

### 4.2 The relationship between attentional bias and psychological craving in MUD individuals

This study found that attentional bias in MUD individuals was significantly positively correlated with both withdrawal craving and cue craving. This finding is consistent with most addiction-related research (26). A meta-analysis showed a strong relationship between attentional bias and craving, which is influenced by various factors (45). Attentional bias and craving are two interrelated psychological phenomena that may share similar underlying mechanisms. Specifically, some studies suggest that attentional bias and psychological craving are causally linked (21). The subjective craving for MA can lead to an attentional bias toward MA-related cues, and this bias, in turn, further increases craving levels in MUD individuals. This process is modulated by multiple factors, including impulsivity, impaired inhibitory control, the deliberate suppression of craving, and the avoidance of drug cues. Additionally, from a classical conditioning perspective, attentional bias can cause MA-related cues to elicit expectations of drug use, further reinforcing attentional bias toward those cues (21). This interrelationship implies that simultaneous intervention targeting both psychological craving and attentional bias may be more effective in guiding clinical interventions.

Attentional bias can be used to differentiate individuals with varying levels of withdrawal craving and cue craving. Specifically, individuals with low withdrawal craving or no cue craving did not show significant differences in reaction times between MA-related and neutral picture stimuli, while those with high withdrawal craving or high cue craving exhibited longer reaction times to MA-related stimuli. Notably, effect sizes in the results indicate that the high-craving group exhibited larger effect sizes compared to the low or no-craving groups. Furthermore, MUD individuals with different withdrawal craving levels demonstrated intergroup differences in attentional bias. Although no statistically significant differences in attentional bias were found across cue craving groups, as illustrated in Figure 2, attentional bias scores increased with higher cue craving levels. This study supports the notion that MUD individuals with different levels of psychological craving exhibit varying degrees of attentional bias. A review by Field et al. suggests that attentional bias is related to the intensity of craving at the moment (26). And in the context of MA withdrawal, this craving represents a withdrawal craving state. Previous research has also shown that nicotine-dependent individuals experiencing tobacco deprivation, those with more intense craving exhibit a stronger correlation between their craving and attentional bias (51, 52). However, a study on attentional bias and subjective craving in MUD individuals found that both those with and without reported craving after cue exposure showed significantly higher reaction times to drug-related cues than to neutral cues. This result differs from our findings, possibly due to variations in cue exposure strength, in the type of attentional bias paradigm or in the method of measurement (53, 54). Furthermore, attentional bias related to illegal substances (e.g., cannabis, cocaine, and heroin) shows a stronger relationship with subjective craving compared to substances like tobacco and alcohol (45).

However, the results further confirm that only withdrawal craving—not cue-induced craving—is independently and specifically associated with attentional bias. It is important to note that the time interval between assessments may also have contributed to the negative results regarding cue-induced craving and attentional bias. Since cue-induced craving is an acute state that decays rapidly over time, the multi-day delay likely weakened any observable relationship. Subsequent studies should aim to minimize this delay by measuring attentional bias immediately after cue exposure within the same experimental session to better capture their dynamic relationship.

These findings also allows us to frame our results within the stage-based model of addiction. Prolonged abstinence does not eliminate the neuroadaptive changes from chronic addiction; instead, it shifts the core motivational driver from acute reward seeking to persistent distress relief. The withdrawal/negative affect stage is defined by negative emotional states (e.g., dysphoria, stress) and weakened reward system function—such as reduced dopamine in the nucleus accumbens (55). Notably, this dopamine deficit may heighten the brain’s prioritization of stimuli linked to distress relief-related stimuli, directing attentional resources automatically toward cues that alleviate withdrawal symptoms and thereby strengthening the aforementioned association. Critically, the context of long-term compulsory abstinence powerfully amplifies this stage. The enforced absence of drugs and drug-related cues suppresses the transient reactivity of the preoccupation/anticipation stage, allowing the persistent, endogenous distress of withdrawal to become the dominant motivational focus. In this environment, attentional bias may thus function primarily as a mechanism of negative reinforcement, aimed at relieving this chronic aversive state.

In contrast, the preoccupation/anticipation stage centers on cue-induced craving and executive function deficits, driven by dysregulation in the prefrontal cortex and insula (15). As cue-induced craving is often triggered by transient external stimuli rather than persistent internal distress, its motivational pull may not be strong enough to consistently shape attentional allocation—may explain why it shows no robust association with attentional bias. Since attentional bias is linked to withdrawal craving rather than cue-induced craving, it logically reflects the cognitive prioritization of alleviating withdrawal-related distress rather than the cue-driven motivational processes (15). This theoretical refinement offers a more precise understanding of when and for whom attentional bias is most salient. Future research could further validate this by examining how attentional bias changes across different stages of addiction and whether stage-specific interventions yield better outcomes.

There are several limitations to this study. First, as a cross-sectional survey, it could not determine the causal relationship between attentional bias and craving. Second, the present study did not include female participants, voluntary MA abstainers, and MUD individuals in short-term abstinence, which limits the generalizability of our findings. Third, the multi-day interval between the assessments of cue-induced craving and attentional bias may have led to the decay of the transient craving state, potentially confounding the observable relationship. In addition, objective physiological data such as electrocardiogram, electrodermal activity, and electroencephalogram were not collected simultaneously. Larger scale, more comprehensive assessments, and longitudinal cohort studies should be conducted to investigate the characteristics of attentional bias among MUD participants across different stages. Specifically, these attentional bias characteristics at different abstinence stages is a critical direction for future research.

## 5 Conclusion

The MUD individuals exhibit a clear attentional bias toward MA-related cues, which is associated with withdrawal craving rather than cue-induced craving. Attentional bias could serve as a cognitive marker of the withdrawal/negative affect stage. Attentional bias can differentiate individuals with varying levels of withdrawal and cue-induced craving, and higher withdrawal craving is linked to more pronounced attentional bias. Moreover, the relationship between attentional bias and psychological craving provides a basis for further intervention research to reduce both.

## Data Availability

The raw data supporting the conclusions of this article will be made available by the authors, without undue reservation.
